# Protocol to purify and detect ubiquitinated phospholipids in budding yeast and human cell lines

**DOI:** 10.1016/j.xpro.2022.101935

**Published:** 2022-12-13

**Authors:** Jun-ichi Sakamaki, Noboru Mizushima

**Affiliations:** 1 Department of Biochemistry and Molecular Biology, Graduate School of Medicine, The University of Tokyo, Tokyo 113-0033, Japan

**Keywords:** Cell Biology, Model Organisms, Molecular Biology, Protein Biochemistry

## Abstract

Ubiquitin is covalently conjugated to phospholipids as well as proteins; however, ubiquitinated phospholipids are less abundant than free ubiquitin and ubiquitinated proteins. Here, we describe protocols to purify ubiquitinated phospholipids in budding yeast and human cells based on their hydrophobicity. Ubiquitinated phospholipids are purified by Triton X-114 phase partitioning and affinity purification and verified by phospholipase D treatment. These protocols enable the detection of tagged as well as endogenous mono- and poly-ubiquitinated phospholipids by immunoblotting.

For complete details on the use and execution of this protocol, please refer to Sakamaki et al..^1^

## Highlights

• Protocol for detection of ubiquitinated PE
• Purification of ubiquitinated PE by Triton X-114 phase partitioning
• Verification of ubiquitin conjugation to phospholipids using phospholipase D
• Detection of purified ubiquitinated PE by immunoblotting

## Abstract (editor highlights)

Publisher’s note: Undertaking any experimental protocol requires adherence to local institutional guidelines for laboratory safety and ethics.

## Graphical abstract

**Figure:**
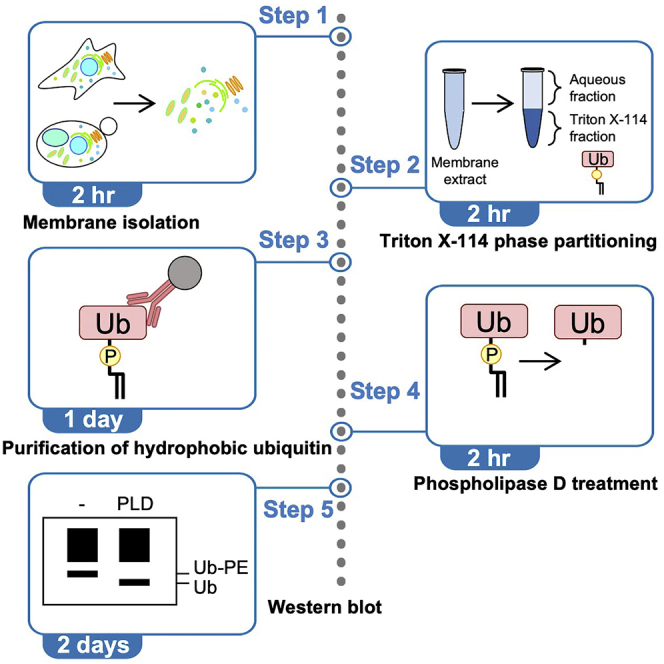

## Before you begin

**Figure 1:**
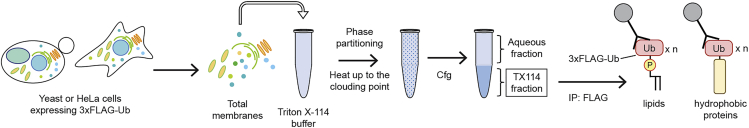
Purification procedure of hydrophobic ubiquitin (Ub) species Total membranes are isolated from yeast or HeLa cells expressing 3×FLAG-Ub and solubilized in a buffer containing 1% Triton X-114. The sample is subjected to Triton X-114 phase partitioning followed by immunoprecipitation of 3×FLAG-Ub in the Triton X-114 fraction. Hydrophobic ubiquitin species contain ubiquitinated phospholipids and hydrophobic proteins (e.g., membrane proteins).

Ubiquitination is a common post-translational protein modification that regulates the stability, localization, activity, and complex formation of target proteins.^2^^,^^3^^,^^4^ We have recently shown that the phospholipid phosphatidylethanolamine (PE) is also ubiquitinated in budding yeast and human cells.^1^ However, because ubiquitinated PE (Ub-PE) is less abundant than free ubiquitin and ubiquitinated proteins, it is difficult to detect Ub-PE by immunoblotting of whole cell lysates. Thus, we describe protocols for the efficient purification of Ub-PE from yeast and human cells. When conjugated to PE, ubiquitin becomes hydrophobic and can be separated by Triton X-114 phase partitioning.^5^^,^^6^^,^^7^^,^^8^ Triton X-114 has a low cloud point of approximately 23°C, and a buffer containing Triton X-114 separates into the detergent and aqueous phases above the cloud point. Hydrophobic proteins, including lipid-conjugated proteins and membrane proteins, are incorporated into the detergent phase owing to their hydrophobicity. On the other hand, free ubiquitin and soluble ubiquitinated proteins, which are abundant in cells, are incorporated into the aqueous fraction and can be removed. Hydrophobic ubiquitin species in the detergent fraction are further purified by affinity purification (Figure 1). Ub-PE is detected at a slightly higher position than that of the unmodified monomeric ubiquitin on an immunoblot. Phospholipase D, which hydrolyzes a phosphodiester bond of glycerophospholipids, causes a downward shift of Ub-PE, validating the conjugation of ubiquitin to PE. Below are the procedures to purify endogenous and tagged Ub-PE from yeast and human cells.

### Yeast cell culture

**Timing: 1 day**

1. To detect 3xFLAG tagged ubiquitinated PE (Ub-PE), collect cells as described below. troubleshooting 1**.**
  a. Inoculate three tubes (e.g., 16-mL culture tube with two-position cap; Evergreen, Cat#: 222-2094-050) of 4 mL of synthetic defined-casamino acid (SD-CA) (-Ura) medium (total 12 mL) with BY4741 cells carrying pRS316-glyceraldehyde-3-phosphate dehydrogenase (GPD) promoter-3×FLAG-Ub and culture at 30°C using a rotator (TAITEC, model# RT-50N; speed, 7).
    **CRITICAL:** 1a should be performed under sterile conditions.
  b. Collect log-phase cells (OD_600_ ≈ 1.0) in a 15-mL tube by centrifugation at 1,000 × *g* for 5 min.
  c. Wash cells with 1 mL of ddH_2_O and proceed to step 1 of the step-by-step method details.
2. To detect endogenous Ub-PE, collect cells as described below.
  a. Inoculate 50 mL of yeast extract–peptone–dextrose (YPD) medium in a 300-mL flask with BY4741 cells and culture with shaking at 150 rpm at 30°C.
    **CRITICAL:** 2a should be performed under sterile conditions.
  b. Collect log-phase cells (OD_600_ ≈ 1.0) in a 50-mL tube by centrifugation at 1,000 × *g* for 5 min.
  c. Wash cells with 5 mL of ddH_2_O and proceed to step 1 of the step-by-step method details.

### Human cell culture

**Timing: 2–3 days**

3. To detect 3xFLAG-Ub-PE, collect cells as described below. troubleshooting 1.
  a. Seed HeLa cells stably expressing 3xFLAG-Ub in DMEM complete medium into two 10-cm dishes.
    **CRITICAL:** 3a–b should be performed under sterile conditions.
  b. Culture cells for 2–3 days until cells reach 80% confluence.
  c. Remove the medium and wash cells with 10 mL of ice-cold phosphate-buffered saline (PBS).
  d. Collect the cells by scraping in 1 mL of ice-cold PBS and transfer them to a 1.5-mL tube.
  e. Centrifuge at 1,000 × *g* at 4°C for 5 min.
  f. Remove the supernatant and proceed to step 2 of the step-by-step method details.
4. To detect endogenous Ub-PE, collect cells as described below.
  a. Seed HeLa cells in DMEM complete medium into ten 10-cm dishes.
    **CRITICAL:** 4a–c should be performed under sterile conditions.
  b. Culture cells for 2–3 days until cells reach 80% confluence.
  c. Remove the medium and wash cells with 10 mL of PBS.
  d. Add 1 mL of ice-cold PBS to each dish, collect the cells by scraping, and transfer them to a 15-mL tube.
  e. Centrifuge at 1,000 × *g* at 4°C for 5 min.
  f. Remove the supernatant and proceed to step 2 of the step-by-step method details.

### Preparation of GST-ubiquilin 1 ubiquitin-associated domain (UQ1 UBA)

**Timing: 4 days**

***Note:*** Glutathione *S*-transferase (GST)-ubiquilin 1 ubiquitin-associated domain (UQ1 UBA) is used to capture endogenous ubiquitin. When 3xFLAG-Ub-PE is detected, this step can be skipped.

5. For the affinity purification of endogenous Ub-PE, prepare GST-UQ1 UBA^9^ as follows. troubleshooting 2**.**
  a. Transform BL21 cells with pGEX6p-UQ1 UBA and culture at 37°C for 12–18 h.
  b. Inoculate 5 mL of 2× yeast extract tryptone (2xYT) + carbenicillin in a 50-mL tube (e.g., Thermo Fisher Scientific, Cat#: 339652) with a colony of the transformant and culture with shaking at 200 rpm at 37°C for 12–18 h.
  c. Transfer the culture to 200 mL of 2xTY + carbenicillin in a 500-mL flask and culture with shaking at 160 rpm at 30°C for 4 h.
  d. Add 100 μL of 1 M isopropyl-β-d(-)-thiogalactopyranoside (IPTG) (final concentration, 0.5 mM) and culture with shaking at 160 rpm at 30°C for 4 h.
  e. Collect cells by centrifugation at 2,900 × *g* for 5 min.
  f. Resuspend cells in 10 mL of ice-cold CelLytic B containing proteinase inhibitor cocktail and 1 mM dithiothreitol (DTT), transfer the cell suspension to a 50-mL tube, and incubate on ice for 15 min.
  g. Sonicate for 10 s, twice (Astrason, model# XL2020; strength, 3.5).
  h. Remove cell debris by centrifugation at 4°C at 17,700 × *g* for 10 min.
  i. Pass the supernatant through a 0.45-μm pore filter and transfer it to a 15-mL tube.
  j. Incubate the supernatant with 1 mL of glutathione Sepharose 4B (50% in CelLytic B buffer) at 4°C for 12–18 h using a rotator.
  k. Wash the beads with 1 mL of ice-cold CelLytic B reagent three times.
  l. Wash the beads with 1 mL of ice-cold Triton X-114 wash/dilution buffer three times.
  m. Store the beads in 500 μL of Triton X-114 wash/dilution buffer (50% in buffer) at 4°C.
  n. To confirm that GST-UQ1 UBA is properly generated, mix 5 μL of GST-UQ1 UBA with 1 μL of 6× SDS-PAGE sample buffer and perform SDS polyacrylamide gel electrophoresis (SDS-PAGE) followed by Coomassie brilliant blue (CBB) staining. GST-UQ1 UBA should be detected at approximately 31 kDa.

***Note:*** To spin down the beads, centrifuge at 4°C at 1,000 × *g* for 1 min.

## Key resources table

**Table:** 

REAGENT or RESOURCE	SOURCE	IDENTIFIER
**Antibodies**		

Mouse monoclonal anti-FLAG (clone M2) (WB: 1/2000)	Sigma-Aldrich	Cat#: F1804; RRID: AB_262044
Mouse monoclonal anti-FLAG (clone M2) affinity gel (IP)	Sigma-Aldrich	Cat#: A2220; RRID: AB_10063035
Mouse monoclonal anti-ubiquitin (clone VU-1) (WB: 1/500)	LifeSensors	Cat#: VU101; RRID: AB_2716558
Rabbit polyclonal anti-LAMP1 (WB: 1/2000)	Abcam	Cat#: ab24170; RRID: AB_775978
Rabbit monoclonal anti-LAMP1 (clone D2D11) (WB: 1/2000)	Cell Signaling Technology	Cat#: 9091; RRID: AB_2687579
Rabbit monoclonal anti-RAB7 (clone D95F2) (WB: 1/2000)	Cell Signaling Technology	Cat#: 9367; RRID: AB_1904103
Mouse monoclonal anti-Pho8 (clone 1D3A10) (WB: 1/2000)	Abcam	Cat#: ab113688; RRID: AB_10860792
Mouse monoclonal anti-Vph1 antibody (clone 10D7A7B2) (WB: 1/2000)	Abcam	Cat#: ab113683; RRID: AB_10863889
Mouse monoclonal anti-Pep12 antibody (clone 2C3G4) (WB: 1/2000)	Abcam	Cat#: ab113689; RRID: AB_10862365
Horseradish peroxidase–conjugated anti-mouse IgG (WB: 1/5000)	Jackson ImmunoResearch Laboratories	Cat#: 315-035-003; RRID: AB_2340061

**Bacterial and virus strains**		

BL21	TAKARA	9126

**Chemicals, peptides, and recombinant proteins**		

Yeast extract	Becton, Dickinson and Company	212750
Bacto Peptone	Becton, Dickinson and Company	211677
Glucose	Nacalai Tesque	16805-35
Yeast nitrogen base without amino acids and ammonium sulfate	Becton, Dickinson and Company	233520
Ammonium sulfate	Nacalai Tesque	02619-15
Casamino acids	Becton, Dickinson and Company	223050
Adenine	Sigma-Aldrich	A3159
L-tryptophan	Fujifilm	204-03382
Sodium hydroxide	Nacalai Tesque	31511-05
Dulbecco’s modified eagle medium	Sigma-Aldrich	D6546
Fetal bovine serum	Sigma-Aldrich	173012
L-glutamine	Thermo Fisher Scientific	25030-081
Torin1	Millipore	475991
Concanamycin A	Abcam	ab144227
Bafilomycin A_1_	Sigma-Aldrich	B1793
Dimethyl sulfoxide (DMSO)	Sigma-Aldrich	D2650
2× yeast extract tryptone (2×YT)	Becton, Dickinson and Company	244020
Carbenicillin	Sigma-Aldrich	C1389
Isopropyl-β-D(-)-thiogalactopyranoside (IPTG)	Fujifilm	099-02534
CelLytic B cell lysis reagent for bacterial cell lysis	Sigma-Aldrich	B7435
Dithiothreitol (DTT)	Nacalai Tesque	14112-52
Glutathione Sepharose 4B	Cytiva	17-0756-01
Sucrose	Fujifilm	196-00015
Sodium chloride	Nacalai Tesque	31319-74
4-(2-hydroxyethyl)-1-piperazineethanesulfonic acid (HEPES)	Nacalai Tesque	17514-15
Tris(hydroxymethyl)aminomethane (Tris)	Nacalai Tesque	35434-34
Potassium chloride	Fujifilm	163-03545
Magnesium chloride	Nacalai Tesque	20908-65
Calcium chloride	Fujifilm	031-00435
Potassium dihydrogen phosphate	Fujifilm	169-04245
Disodium hydrogen phosphate	Nacalai Tesque	31726-05
Triton X-114	Nacalai Tesque	35522-45
Protease inhibitor cocktail	Nacalai Tesque	03963-34
Sodium dodecyl sulfate (SDS)	Nacalai Tesque	02873-75
Glycerol	Fujifilm	075-00616
Bromophenol blue	Fujifilm	021-02911
Phospholipase D	Enzo	BML-SE301
Coomassie brilliant blue (CBB) staining kit	Apro Science	SP-4011
Glutaraldehyde	TAAB Laboratories Equipment	G018/1
Skim milk	Fujifilm	198-10605
Polyoxyethylene sorbitan monolaurate (Tween 20)	Nacalai Tesque	28353-85
Bovine serum albumin	Fujifilm	011-27055
Poly(vinylidene fluoride) (PVDF) membrane	Merck	ISEQ00010
Immobilon Western chemiluminescent horseradish peroxidase substrate	Merck	P90715

**Experimental models: Cell lines**		

HeLa	RIKEN	RCB0007

**Experimental models: Organisms/strains**		

Yeast BY4741 strain	Gilda et al.^10^	N/A

**Recombinant DNA**		

pRS316-GPD promoter-3×FLAG-scUbiquitin (Met1-Gly76)	Sakamaki et al.^1^	N/A
pMRX-IPU-3×FLAG-hUbiquitin (Gln2-Gly76)	Sakamaki et al.^1^	N/A
pCG-gag-pol	Dr. Teruhito Yasui (National Institutes of Biomedical Innovation, Health and Nutrition, Japan)	N/A
pCG-VSVG	Dr. Teruhito Yasui (National Institutes of Biomedical Innovation, Health and Nutrition, Japan)	N/A
pGEX6p-ubiquilin 1 UBA	Sakamaki et al.^1^	N/A

## Materials and equipment

**Table:** Yeast extract–peptone–dextrose (YPD) medium

Reagent	Final concentration	Amount
Yeast extract	1%	10 *g*
Bacto peptone	2%	20 *g*
ddH_2_O	N/A	Up to 900 mL
Autoclaved 20% glucose	2%	100 mL
**Total**	**N/A**	**1 L**

**CRITICAL:** Glucose should be separately prepared as a solution and autoclaved. Glucose solution should be added to the medium under sterile conditions.

Dissolve yeast extract and bacto peptone in 900 mL of ddH2O and autoclave the mixture.

Dissolve 20 *g* of glucose in 100 mL of ddH2O and autoclave the solution.

Add 100 mL of autoclaved 20% glucose to 900 mL of the autoclaved yeast extract and bacto peptone mixture.

Store at 22°C–25°C. Stable for at least a year.

**Table:** Synthetic defined (SD)/casamino acid (CA) (-Ura) medium, pH 6.2

Reagent	Final concentration	Amount
Yeast nitrogen base without amino acids and ammonium sulfate	0.17%	1.7 *g*
Ammonium sulfate	0.5%	5 *g*
Casamino acids	0.5%	5 *g*
5 N NaOH	N/A	750 μL
ddH_2_O	N/A	Up to 880 mL
Autoclaved 20% glucose	2%	100 mL
2 mg/mL of adenine	0.02 mg/mL	10 mL
2 mg/mL of l-tryptophan	0.02 mg/mL	10 mL
**Total**	**N/A**	**1 L**

**CRITICAL:** Glucose, adenine, and l-tryptophan should be separately prepared as solutions and sterilized. Glucose solution and the supplements should be added to the medium under sterile conditions.

Dissolve yeast nitrogen base, ammonium sulfate, and casamino acids in 880 mL of ddH2O, add 750 μL of 5 N NaOH, and autoclave the mixture.

Dissolve 20 g of glucose in 100 mL of ddH2O and autoclave the solution.

Dissolve 200 mg of adenine in 100 mL of ddH2O and sterilize the solution using a 0.22-μm pore filter.

Dissolve 200 mg of l-tryptophan in 100 mL of ddH2O and sterilize the solution using a 0.22-μm pore filter.

Add 100 mL of autoclaved 20% glucose, 10 mL of 2 mg/mL adenine, and 10 mL of 2 mg/mL l-tryptophan to 880 mL of the autoclaved yeast nitrogen base, ammonium sulfate, and casamino acids mixture.

Store at 22°C–25°C. Stable for at least a year.

**Table:** 1 mM Torin1

Reagent	Final concentration	Amount
Torin1	1 mM	10 mg
DMSO	N/A	16.45 mL
**Total**	**N/A**	**16.45 mL**

Make 1-mL aliquots.

Store at −20°C. Stable for at least a year.

**Table:** 500 μM Concanamycin A

Reagent	Final concentration	Amount
Concanamycin A	500 μM	25 μg
DMSO	N/A	57.73 μL
**Total**	**N/A**	**57.73 μL**

Store at −20°C. Stable for at least a year.

**Table:** 100 μM Bafilomycin A_1_

Reagent	Final concentration	Amount
Bafilomycin A_1_	100 μM	10 μg
DMSO	N/A	160.55 μL
**Total**	**N/A**	**160.55 μL**

Store at −20°C. Stable for at least a year.

**Table:** 1 M Dithiothreitol (DTT)

Reagent	Final concentration	Amount
Dithiothreitol	1 M	1.54 *g*
ddH_2_O	N/A	Up to 10 mL
**Total**	**N/A**	**10 mL**

Make 1-mL aliquots.

Store at −20°C. Stable for at least a year.

**Table:** 1 M Isopropyl-β-d(-)-thiogalactopyranoside (IPTG)

Reagent	Final concentration	Amount
Isopropyl-β-d(-)-thiogalactopyranoside	1 M	2.38 *g*
ddH_2_O	N/A	Up to 10 mL
**Total**	**N/A**	**10 mL**

Make 1-mL aliquots.

Store at −20°C. Stable for at least a year.

**Table:** 2× yeast extract tryptone (2xYT) + carbenicillin

Reagent	Final concentration	Amount
2× yeast extract tryptone	N/A	15.5 *g*
ddH_2_O	N/A	Up to 500 mL
**Total**	**N/A**	**500 mL**

Autoclave 2xYT medium.

Add 500 μL of 50 mg/mL carbenicillin (sterilized using a 0.22-μm pore filter) (final concentration, 50 μg/mL).

Store at 4°C. Stable for several months.

**Table:** Dulbecco’s modified eagle medium (DMEM) complete medium

Reagent	Final concentration	Amount
DMEM	N/A	500 mL
Fetal bovine serum	approximately 10 %	55 mL
l-glutamine	approximately 2 mM	5.5 mL
**Total**	**N/A**	**560.5 mL**

**CRITICAL:** The medium should be prepared under sterile conditions.

Store at 4°C. Stable for several months.

**Table:** Disruption buffer

Reagent	Final concentration	Amount
1 M Tris-HCl (pH 7.5)	10 mM	500 μL
5 M NaCl	150 mM	1.5 mL
1 M sucrose	250 mM	12.5 mL
ddH_2_O	N/A	35.5 mL
**Total**	**N/A**	**50 mL**

Store at 4°C. Stable for at least a year.

**Table:** Homogenization buffer

Reagent	Final concentration	Amount
1 M Tris-HCl (pH 7.5)	10 mM	500 μL
3 M KCl	10 mM	166.66 μL
1 M MgCl_2_	1.5 mM	75 μL
1 M sucrose	250 mM	12.5 mL
ddH_2_O	N/A	36.75 mL
**Total**	**N/A**	**50 mL**

Store at 4°C. Stable for at least a year.

**Table:** Triton X-114 buffer

Reagent	Final concentration	Amount
1 M Tris-HCl (pH 7.5)	10 mM	500 μL
5 M NaCl	150 mM	1.5 mL
10% Triton X-114	1 %	5 mL
ddH_2_O	N/A	42.5 mL
**Total**	**N/A**	**49.5 mL**

Add 1/100 volume of 100x protease inhibitor cocktail before use.

Store at 4°C. Stable for at least a year.

**Table:** Triton X-114 wash/dilution buffer

Reagent	Final concentration	Amount
1 M Tris-HCl (pH 7.5)	10 mM	500 μL
5 M NaCl	150 mM	1.5 mL
10% Triton X-114	0.06 %	300 μL
ddH_2_O	N/A	47.7 mL
**Total**	**N/A**	**50 mL**

Store at 4°C or 22°C–25°C. Stable for at least a year.

**Table:** Phospholipase D (PLD) buffer

Reagent	Final concentration	Amount
1 M Hepes-KOH (pH 8.0)	40 mM	2 mL
1 M CaCl_2_	4 mM	200 μL
ddH_2_O	N/A	47.8 mL
**Total**	**N/A**	**50 mL**

Store at 4°C. Stable for at least a year.

**Table:** 6× sample buffer

Reagent	Final concentration	Amount
1 M Tris-HCl (pH6.8)	0.3 M	15 mL
SDS	0.12 *g*/mL	6 *g*
Glycerol	60 %	30 mL
Bromophenol blue	0.6 mg/mL	30 mg
ddH_2_O	N/A	Up to 45 mL
**Total**	**N/A**	**45 mL**

Make 0.9-mL aliquots.

Store at −20°C. Stable for at least a year.

Add 100 μL of 1 M DTT (final concentration, 0.1 M) to the aliquot before use.

After the addition of DTT, store at 22°C–25°C. Stable for at least several months.

**Table:** 25× phosphate-buffered saline (25xPBS)

Reagent	Final concentration	Amount
NaCl	3.425 M	200 *g*
Na_2_HPO_4_	202.5 mM	28.75 *g*
KCl	67.5 mM	5 *g*
KH_2_PO_4_	36.75 mM	5 *g*
ddH_2_O	N/A	Up to 1 L
**Total**	**N/A**	**1 L**

To make 1xPBS, dilute 40 mL of 25xPBS with 960 mL of ddH_2_O.

Store at 22°C–25°C. Stable for at least a year.

**Table:** 25× Tris-buffered saline (25xTBS)

Reagent	Final concentration	Amount
NaCl	3.425 M	200 *g*
Tris	500 mM	60.5 *g*
ddH_2_O	N/A	Up to 1 L
**Total**	**N/A**	**1 L**

Adjust pH to 7.6 with HCl.

To make 1xTBS, dilute 40 mL of 25xTBS with 960 mL of ddH2O.

To make TBST, add 1 mL of Tween-20 to 1xTBS (final concentration, 0.1%).

Store at 22°C–25°C. Stable for at least a year.

## Step-by-step method details

### Preparation of total membranes from yeast and human cells

**Timing: 2 h**

This section describes the isolation of total membranes from 12-mL cultures of budding yeast and two 10-cm dishes of HeLa cells. To detect endogenous Ub-PE in 50 mL of yeast culture or ten 10-cm dishes of HeLa cells, use 5× volumes of buffers throughout the procedures described below.

1. Isolation of total membrane from yeast cells.
  a. Resuspend cells in 500 μL of ice-cold disruption buffer containing protease inhibitor cocktail.
  b. Transfer the cell suspension to a 2-mL tube (e.g., Watson Bio Lab, Cat# 1392-200) containing approximately 500 μL of 0.5 mm zirconium dioxide beads.
  c. Disrupt cells using a multi-beads shocker (Yasui Kikai, 2700 rpm, 30 s on/30 s off, twice).
  d. Remove intact cells, cell debris, and nuclei by centrifugation at 1,000 × *g* at 4°C for 5 min.
  e. Collected 25 μL of the supernatant and mix it with 5 μL of 6× sample buffer (total lysate).
  f. Apply the supernatant to ultracentrifugation at 100,000 × *g* at 4°C for 1 h. troubleshooting 3.
  g. Remove the supernatant (the cytosol) and proceed to step 3.

***Note:*** To efficiently recover the lysate at step d, puncture the bottom of the 2-mL tube using an 18-gauge needle, place it in a 15-mL tube, and carry out centrifugation.

2. Isolation of total membrane from HeLa cells.
  a. Resuspend cells in 500 μL of ice-cold homogenization buffer.
  b. Centrifuge at 4°C at 1,000 × *g* for 5 min.
  c. Remove the supernatant, resuspend cells in 500 μL of ice-cold homogenization buffer containing protease inhibitor cocktail, and incubate on ice for 10 min.
  d. Disrupt cells using a 27-gauge needle and syringe unit (20 strokes).
  e. Centrifuge at 4°C at 1,000 × *g* for 5 min to remove intact cells, cell debris, and nuclei.
  f. Collect 25 μL of the supernatant and mix it with 5 μL of 6× sample buffer (total lysate).
  g. Apply the supernatant to ultracentrifugation at 100,000 × *g* at 4°C for 1 h. troubleshooting 3.
  h. Remove the supernatant (i.e., the cytosol) and proceed to step 3.

***Note:*** Cells can also be disrupted by nitrogen decompression (Parr Instrument Company, model# 4639 Cell Disruption Vessel, 500 psi).

### Purification of hydrophobic proteins

**Timing: 2 h**

This section describes the purification of hydrophobic proteins by Triton X-114 phase partitioning. When performing the delipidation assay for verification of conjugation of ubiquitin to phospholipids, proceed to step 7 after completing this step (see also step 7 for details).

3. Purification and concentration of hydrophobic ubiquitin.
  a. Resuspend the pellet (i.e., membranes) in 200 μL of ice-cold Triton X-114 buffer containing protease inhibitor cocktail.
  b. Centrifuge at 4°C at 16,900 × *g* for 10 min to remove the insoluble fraction.
  c. Transfer the supernatant to a new 1.5-mL tube.
  d. Place the tube at 37°C for approximately 1 min (until the solution becomes cloudy).
  e. Centrifuge at 25°C at 16,900 × *g* for 10 min.
  f. Remove the top layer (i.e., the aqueous phase, approximately 180 μL in the first round and 500 μL in the wash steps).
  g. Add 500 μL of Triton X-114 wash/dilution buffer and mix well.
  h. Place the tube on ice until the solution becomes clear (within several min).
  i. Repeat steps 3d–3h twice (for a total of three repetitions of wash steps). troubleshooting 4.
  j. After the third repetition of wash steps, add 500 μL of Triton X-114 wash/dilution buffer containing a proteinase inhibitor cocktail to the detergent phase.
  k. Place the tube on ice until the solution becomes clear (within several min).
  l. Centrifuge at 4°C at 16,900 × *g* for 10 min to remove debris.
  m. Transfer approximately 520 μL of the supernatant to a new 1.5-mL tube.
  n. Proceed to step 4 or 5.

***Note:*** Buffers containing Triton X-114 separate into the detergent and aqueous phases above the cloud point (approximately 23°C) and become cloudy.

### Purification of hydrophobic ubiquitin

**Timing: 1 day**

This section describes the purification of hydrophobic ubiquitin by immunoprecipitation with anti-FLAG antibody (step 4) and pull-down with ubiquilin 1 ubiquitin-associated domain (UQ1 UBA)^9^ (step 5).

4. Immunoprecipitation of 3xFLAG-Ub with anti-FLAG antibody
  a. Wash anti-FLAG M2 affinity beads with 1 mL of Triton X-114 wash/dilution buffer three times.
  b. Add 20 μL of anti-FLAG M2 affinity beads (50% in Triton X-114 wash/dilution) to the sample.
  c. Incubate at 4°C for 12–18 h using a rotator.
  d. Wash the beads with 500 μL of Triton X-114 wash/dilution buffer three times.
  e. Wash the beads with 500 μL of ddH_2_O twice.
  f. Proceed to step 6 or add 50 μL of 1× sample buffer and proceed to step 8.

***Note:*** To spin down the beads, centrifuge at 4°C at 1,000 × *g* for 1 min.

**Pause point:** After mixing with 1× sample buffer, the samples can be stored at −20°C until until proceeding to SDS-PAGE and immunoblotting.

5. Purification of endogenous ubiquitin using GST-UQ1 UBA
  a. Add 20 μL of GST-UQ1 UBA immobilized on glutathione Sepharose (50% in Triton X-114 wash/dilution buffer).
  b. Incubate at 4°C for 12–18 h using a rotator.
  c. Wash the beads with 500 μL of Triton X-114 wash/dilution buffer three times.
  d. Wash the beads with 500 μL of ddH_2_O twice.
  e. Proceed to step 6 or add 50 μL of 1× sample buffer and proceed to step 8.

***Note:*** UQ1 UBA preferentially captures oligo- and poly-ubiquitin rather than mono-ubiquitin.

**Pause point:** After mixing with 1× sample buffer, the samples can be stored at −20°C until until proceeding to SDS-PAGE and immunoblotting.

### Verification of conjugation of ubiquitin to phospholipids by phospholipase D

**Timing: 1.5–2.5 h**

**Figure 2:**
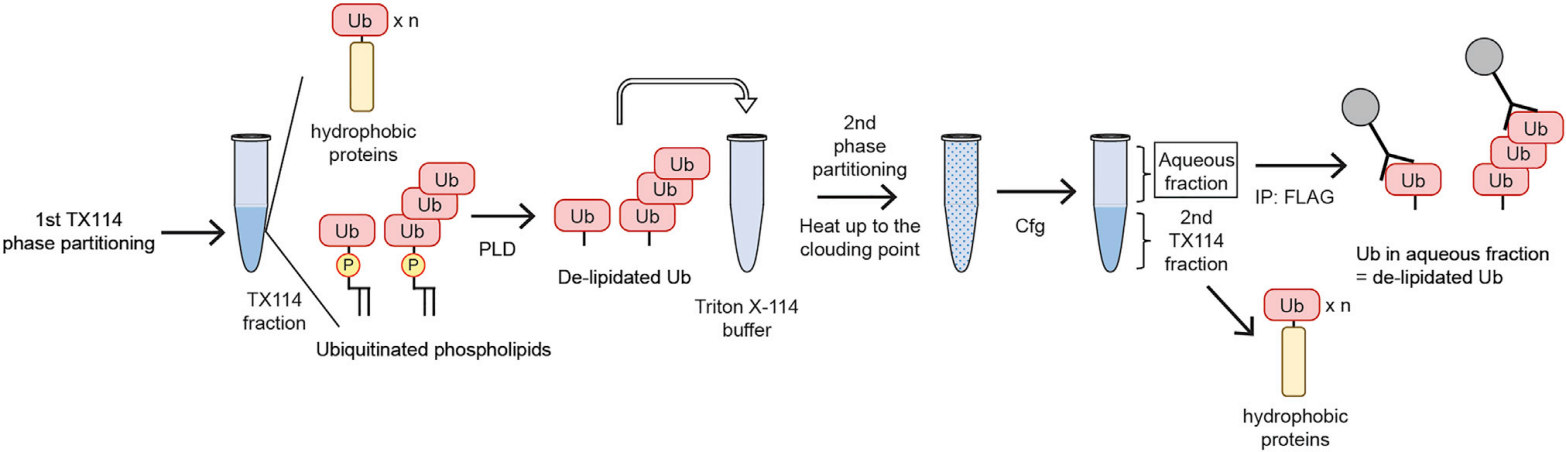
Procedure of the delipidation assay The Triton X-114 fraction is incubated with phospholipase D (PLD) followed by another round of phase partitioning. Delipidated ubiquitin (Ub) species migrate from the Triton X-114 to the aqueous fraction, while hydrophobic proteins remain in the Triton X-114 fraction.

To verify that ubiquitin is conjugated to phospholipids, the sensitivity of hydrophobic ubiquitin to phospholipase D (PLD) can be tested. This section describes on-bead PLD treatment and a delipidation assay using the Triton X-114 fraction in step 3. In the delipidation assay, the purified Triton X-114 fraction is incubated with PLD and subjected to another round of phase partitioning (Figure 2). After PLD treatment, delipidated ubiquitin species are remobilized from the Triton X-114 to the aqueous fraction, while hydrophobic proteins (e.g., ubiquitinated membrane proteins) remain in the Triton X-114 fraction.

6. Treatment of immunoprecipitated hydrophobic ubiquitin with phospholipase D.
  a. Wash the beads with 200 μL of PLD buffer.
  b. Add 50 μL of 1 unit/μL PLD in PLD buffer to beads.
  c. Incubate at 30°C with shaking at 1200 rpm for 1 h.
  d. Wash the beads with 200 μL of ddH_2_O.
  e. Add 50 μL of 1× sample buffer and proceed to step 8.

**Pause point:** After mixing with 1× sample buffer, the samples can be stored at −20°C until until proceeding to SDS-PAGE and immunoblotting.

7. Delipidation assay.
  a. Perform Triton X-114 phase partitioning as described in step 3.
  b. After the third repetition of wash steps, add 500 μL of PLD buffer instead of Triton X-114 wash/dilution buffer to the detergent phase.
  c. Add PLD at 1 unit/μL.
  d. Incubate at 30°C for 1 h.
  e. Centrifuge at 25°C at 16,900 × *g* for 10 min.
  f. Transfer approximately 500 μL of the top layer (the aqueous phase) to a new 1.5-mL tube and add 5 μL of 100x protease inhibitor cocktail.
  g. Add 500 μL of Triton X-114 wash/dilution buffer to the bottom layer (i.e., the detergent phase).
  h. Place the tube on ice until the solution becomes clear (within a couple of min).
  i. Place the tube at 37°C for approximately 1 min (until the solution becomes cloudy).
  j. Centrifuge at 25°C at 16,900 × *g* for 10 min.
  k. Remove the top layer.
  l. Add 500 μL of Triton X-114 wash/dilution buffer containing protease inhibitor cocktail.
  m. Centrifuge at 4°C at 16,900 × *g* for 10 min to remove debris.
  n. Transfer the supernatant to a new 1.5-mL tube.
  o. Purify ubiquitin from the samples in steps 7f and 7n as described in step 4 or 5.

### SDS-PAGE and immunoblotting

**Timing: 2 days**

This section describes the detection of Ub-PE by immunoblotting. To detect endogenous ubiquitin, we use anti-ubiquitin monoclonal antibody (clone VU-1), which sensitively detects mono- and poly-ubiquitin.^10^ This antibody requires pretreatment of a transferred membrane with 0.5% glutaraldehyde.

8. SDS-PAGE.
  a. Load 2 μL of the total lysates and 15 μL of the affinity-purified samples and run them on a 15% gel. troubleshooting 5.
  b. Transfer proteins to a poly (vinylidene fluoride) (PVDF) membrane with 0.2-μm pore size.
9. Immunoblotting.
  a. Detection of 3xFLAG-Ub by anti-FLAG antibody.
    i. Incubate the membrane in 5% skim milk/Tris buffered saline with Tween 20 (TBST) for 1 h.
    ii. Incubate the membrane with anti-FLAG (dilution, 1/2000) in 5% bovine serum albumin (BSA)/TBST at 4°C for 12–18 h and proceed to step 9c.
  b. Detection of endogenous ubiquitin by anti-Ub antibody.
    i. Wash the PVDF membrane with PBS for 2 min three times.
    ii. Incubate the membrane in 0.5% glutaraldehyde in PBS at 22°C–25°C for 20 min.
    iii. Wash the membrane with PBS three times.
    iv. Incubate the membrane in 5% skim milk/TBST for 1 h.
    v. Incubate the membrane with anti-Ub (dilution, 1/500) in 5% BSA/TBST at 4°C for 12–18 h and proceed to step 9c.
  c. Incubation with a secondary antibody and detection.
    i. Wash the membrane with TBST for 5 min three times.
    ii. Incubate the membrane with horseradish peroxidase-conjugated anti-mouse IgG (dilution, 1/5000) in 5% skim milk/TBST 22°C–25°C for 1 h.
    iii. Wash the membrane with TBST for 5 min four times.
    iv. Develop using Immobilon western chemiluminescent horseradish peroxidase substrate.
    v. Acquire images using a Fusion Solo 7S system (Vilber).

## Expected outcomes

**Figure 3:**
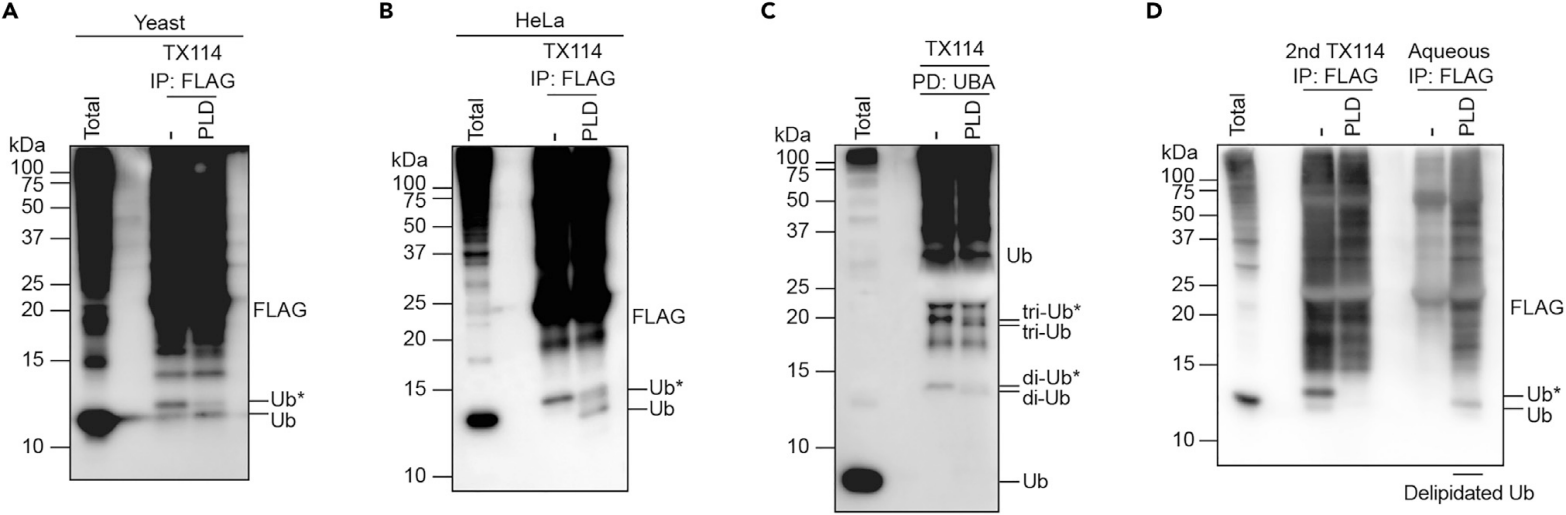
Detection of ubiquitinated phosphatidylethanolamine (Ub-PE) (A and B) Hydrophobic ubiquitin (Ub) species from the Triton X-114 fraction of BY4741 (A) or HeLa (B) cells expressing 3×FLAG-Ub were treated with phospholipase D (PLD). (C) Endogenous hydrophobic ubiquitin species were purified from yeast cells using GST-UQ1 UBA and were treated with PLD. (D) A delipidation assay was performed using the Triton X-114 fraction prepared from yeast cells expressing 3×FLAG-Ub.

Higher molecular weight ubiquitin monomers are detected in the Triton X-114 fraction. This band moves downwards upon phospholipase D treatment (Figures 3A–3C).

In the delipidation assay, mono- and poly-ubiquitinated ubiquitin species move from the Triton X-114 fraction to the aqueous phase upon phospholipase D treatment (Figure 3D).

## Limitations

A large amount of poly-ubiquitinated species is detected in the Triton X-114 fraction. However, it is difficult to assess whether these species are poly-ubiquitinated PE, because ubiquitinated membrane proteins are also enriched in the Triton X-114 fraction. To detect poly-ubiquitinated PE, perform the delipidation assay as described in step 7.

Ub-PE is more sensitive to antibodies compared to unmodified ubiquitin as also reported for ATG8.^11^^,^^12^^,^^13^ It is therefore difficult to accurately estimate the percentage of Ub-PE by this method.

## Troubleshooting

### Problem 1

Weak Ub-PE signal (yeast and human cell culture in before you begin).

### Potential solution

Although Ub-PE is detected under basal conditions, its level is not high. To address this, increase the cell number or use a stimulus, such as nitrogen starvation (3 h) and concanamycin A (500 nM, 3 h) (yeast cells) or Torin 1 (500 nM, 24 h) and bafilomycin A_1_ (100 nM, 6 h) (human cells), to increase Ub-PE levels.

### Problem 2

Low efficiency in ubiquitin pull-down (preparation of GST-UQ1 UBA in before you begin).

### Potential solution

Check that GST-UQ1 UBA is properly produced by conducting SDS-PAGE and CBB staining. The typical yield is approximately 1–2 μg/μL.

### Problem 3

Weak or no Ub-PE signal (isolation of total membrane from yeast and HeLa cells in steps 1 and 2).

### Potential solution

Check that membranes are retrieved after ultracentrifugation. To do this, add 50 μL of 1× sample buffer to the pellet and perform immunoblotting. It is important to confirm that endosomal and vacuolar (or lysosomal) markers are detected in the membrane fraction, because ubiquitinated PE is mainly localized to these organelles.^1^ The following antibodies can be used: rabbit polyclonal anti-LAMP1 (Abcam, Cat#: ab24170, use at 1/2000 dilution), rabbit monoclonal anti-LAMP1 (clone D2D11) (Cell signaling technology, Cat#: 9091, use at 1/2000 dilution), rabbit monoclonal anti-RAB7 (clone D95F2) (Cell signaling technology, Cat#: 9367, use at 1/2000 dilution), mouse monoclonal anti-Pho8 (clone 1D3A10) (Abcam, Cat#: ab113688, use at 1/2000 dilution), mouse monoclonal anti-Vph1 (clone 10D7A7B2) (Abcam, Cat# ab113683, use at 1/2000 dilution), and mouse monoclonal anti-Pep12 antibodies (clone 2C3G4) (Abcam, Cat#: ab113689, use at 1/2000 dilution).

### Problem 4

Contamination of soluble proteins (purification of hydrophobic proteins in step 3).

### Potential solution

Increase the repetitions of the wash steps.

### Problem 5

No clear molecular weight shift of Ub-PE (SDS-PAGE in step 8).

### Potential solution

Use 15% gel and run the samples longer for better separation among low molecular weight species.

## Resource availability

### Lead contact

Further information and requests for resources and reagents should be directed to and will be fulfilled by the lead contact, Noboru Mizushima (nmizu@m.u-tokyo.ac.jp).

### Materials availability

This study did not generate new unique reagents.

## Data Availability

This study did not generate/analyze datasets/code.
